# Physics-regularized neural networks for predictive modeling of silicon carbide swelling with limited experimental data

**DOI:** 10.1038/s41598-024-78037-7

**Published:** 2024-12-28

**Authors:** Kazuma Kobayashi, Syed Bahauddin Alam

**Affiliations:** 1 Nuclear, Plasma & Radiological Engineering, University of Illinois Urbana-Champaign, Urbana, USA; 2 National Center for Supercomputing Application, University of Illinois Urbana-Champaign, Urbana, USA

**Keywords:** Engineering, Energy science and technology, Nuclear energy

## Abstract

This study introduces a physics-regularized neural network (PRNN) as a computational approach to predict silicon carbide’s (SiC) swelling under irradiation, particularly at high temperatures. The PRNN model combines physics-based regularization with neural network methodologies to generalize the behavior of SiC, even in conditions beyond the traditional empirical model’s valid range. This approach ensures continuity and accuracy in SiC behavior predictions in extreme environments. A key aspect of this research is using nested cross-validation to ensure robustness and generalizability. The PRNN model effectively bridges empirical and sparse experimental data by integrating prior knowledge and refined tuning procedures. It demonstrates its SiC’s predictive power in high-irradiation conditions essential for nuclear and aerospace applications.

## Introduction

Silicon carbide (SiC) material can be used in the under extreme conditions, showcasing exceptional thermal, mechanical, and electrical properties^1–3^. Its superior thermal conductivity and structural strength have the potential to be effectively implemented in a wide variety of fields. At the same time, its resistance to radiation makes it invaluable within harsh environments such as nuclear reactors. However, irradiation-induced changes in material properties, particularly swelling, remain a key challenge that can critically undermine material integrity and performance.

In nuclear technology, the deployment of SiC as a cladding material in fuel rods can enhance the efficiency and safety of reactors^4–7^. SiC’s resistance to high temperatures and corrosive fission products leads to higher burnup rates and extended fuel life. However, the volume changes induced by neutron irradiation present a critical issue, potentially impacting material reliability and, by extension, the entire operation of a nuclear reactor. The ability to predict and control these irradiation-induced changes is not just of academic interest but essential for the safe and effective deployment of SiC in nuclear reactors.

In addition to nuclear technology, SiC’s robustness is harnessed in aerospace engineering for structural components within spacecraft and jet engines, where stability under thermal and mechanical stress is paramount^8,9^. However, irradiation can induce swelling, compromising the structural integrity required in high-performance aerospace applications. This presents a significant challenge for advancing aerospace technologies. Similarly, in the burgeoning field of semiconductor devices, SiC’s superior properties allow for devices that operate at higher temperatures and voltages compared to traditional silicon. Yet, irradiation-induced swelling can compromise the semiconductor’s crystalline structure, affecting its electrical properties and overall device performance^10,11^. There is a need across these fields to develop models capable of quantitatively evaluating SiC volume swelling under irradiation. Previous empirical swelling studies^12–14^ formulated models as functions of irradiation temperature and neutron fluence. However, they face significant limitations in high-temperature regimes exceeding 713 K, where irradiation-induced swelling becomes more complex and the empirical models fail to predict behavior accurately. While 713 K represents the normal operating temperature range for conventional light water reactors^15–17^, it is not sufficient for simulating the more severe operating environments of accident conditions or next-generation reactors such as Fast Modular Reactors (FMR)^18–21^. These reactors operate at much higher temperatures, ranging from 1300 to 1500 K in normal conditions, and can experience temperatures up to 2300 K under accident conditions^18^. Therefore, there is a critical research gap in extending models to cover these high-temperature regimes, which are vital for the practical design and development of future nuclear systems. To address these challenges, this study introduces a physics-regularized neural network (PRNN) approach focusing on the unique complexities of irradiation-induced material property changes, particularly swelling under high-temperature irradiation. By combining the available empirical model^13^ with sparse high-temperature experimental data, the PRNN framework bridges the gap between empirical knowledge and the need for accurate predictions in extreme conditions. This method integrates known physical relationships where reliable while allowing for data-driven learning in regions of uncertainty, such as temperatures exceeding 713 K. The use of PRNN offers flexibility by incorporating physical constraints derived from empirical models only in temperature ranges where sufficient data is available, ensuring consistency with known behavior. In higher-temperature regimes, where experimental data is sparse, the regularization is deactivated, allowing the model to generalize based on the available data while mitigating overfitting risks. The strategy of combining known knowledge with machine learning has unfolded through the emergence of Physics-Informed Neural Networks (PINNs)^22^. PINNs inherently encode known physical relationships within the neural network design. By embedding the governing equations and boundary conditions into the loss function and using automatic differentiation, PINNs ensure agreement with the laws of physics across the entire input domain during training^22^. However, adapting such methods^22,23^ to material modeling-where irradiation effects drive complex material behavior may present challenges, particularly when governing physics is incomplete, unclear,  or experimentally confirmed only in specific conditions. The PRNN approach aims to apply a selective regularization, allowing it to generalize a material behavior where physics (or an empirical model available) and ensure continuity with new data obtained in new conditions that exceed their valid range. 

In this paper, the sequence of modeling methods proposed here, combining PRNN, cross-validation, and ensemble methods, provides a pathway to reliably bridge empirical and sparse experimental data through integrating prior knowledge and tuning procedures. This integrated approach, focused on irradiation-induced changes in material properties, offers improved generalization and robustness in predicting material behavior under extreme conditions, contributing to advancements in nuclear and aerospace technologies.

## Methods

### Data augmentation

The primary dataset used in this study was derived from the previous research by Snead et al.^14^, which documents 58 unique entries of the volumetric swelling behavior of SiC under specific irradiation conditions. These conditions span temperatures from 1200 to 1873 K, with displacement damage per atom  (dpa) levels set at 1.75, 5, and 8.5. However, the limited size of this dataset posed challenges for the model’s generalization, particularly in predicting behavior at high temperatures. To address this limitation, a data augmentation strategy was implemented using the k-nearest neighbors (KNN) algorithm from the scikit-learn library^24,25^, generating synthetic data points to enhance the variability and coverage of the dataset. As a first step, the features of the original dataset were normalized using min–max scaling, which transformed all data points to a range between 0 and 1. This normalization ensured that the KNN algorithm operated on a uniform scale, thereby improving the quality of the generated synthetic data. KNN, a non-parametric machine learning algorithm, is used for classification or regression problems; however, it can also be employed for data augmentation by generating synthetic data points based on relationships between existing data points in the feature space^26^. The algorithm identifies the k-nearest neighbors of a given point based on a distance metric (in this case, Euclidean distance), with the proximity of these neighbors providing a foundation for generating new, realistic data points.

For this study, the KNN-based augmentation process was initiated by randomly selecting a seed point from the normalized dataset. The algorithm then identified its five nearest neighbors ($k=5$), representing data points most similar to the seed point in the feature space. These neighboring data points were used to estimate the general trend or structure around the seed point. After identifying the five nearest neighbors, their attributes were averaged to create a base synthetic point. Gaussian noise with a standard deviation of 0.10 was added to the averaged attributes to introduce realistic variability and avoid overfitting. This addition of noise ensured that the generated synthetic points were not mere duplications of existing data but instead represented plausible variations that reflected the natural fluctuations and uncertainties in experimental data. The KNN-based augmentation process was iteratively repeated by selecting different seed points and generating new synthetic data points from their respective neighbors. This iterative process was carried out until 10,000 synthetic data points were generated. By utilizing KNN, the augmented dataset was constructed to maintain the overall structure of the original data while expanding the dataset to capture a wider range of variability. In addition to augmenting the dataset with synthetic data, predictions from an empirical swelling model (Eq. 1) were incorporated to further enrich the data, particularly in temperature ranges where experimental data were sparse. For each dpa level, ten temperatures uniformly sampled between 493 and 1273.15 K were used, and the empirical model was employed to predict the corresponding swelling values. These predictions were then integrated into the augmented dataset, resulting in a combined set of 22,000 data points.

The dataset was expanded by combining KNN-based synthetic data generation with empirical swelling predictions. The resulting augmented dataset spans a broader range of temperatures and dpa levels, providing a more variation for training the PRNN model. This approach improves the model’s ability to generalize across various irradiation scenarios, including high-temperature regions where empirical data are sparse.

### Implementation of PRNN and nested-cross validation

The development of the PRNN was conducted using PyTorch version 1.13.1^27^. PyTorch, with its dynamic computational graph, provided the necessary flexibility for implementing the complex neural network architecture. In parallel, the nested-cross validation (NCV) process was set up using the scikit-learn library^24^, a tool chosen for ensuring a model robustness. NCV, critical for preventing overfitting, incorporates an outer loop for model performance evaluation and an inner loop for hyperparameter tuning. Hyperparameter optimization was carried out using Optuna^28^. 

This approach encompassing the use of PyTorch for PRNN development, scikit-learn for nested cross-validation, and Optuna for hyperparameter optimization, ensured a robust and physically consistent predictive model. 

## Results and discussions

### Empirical model

**Figure 1:**
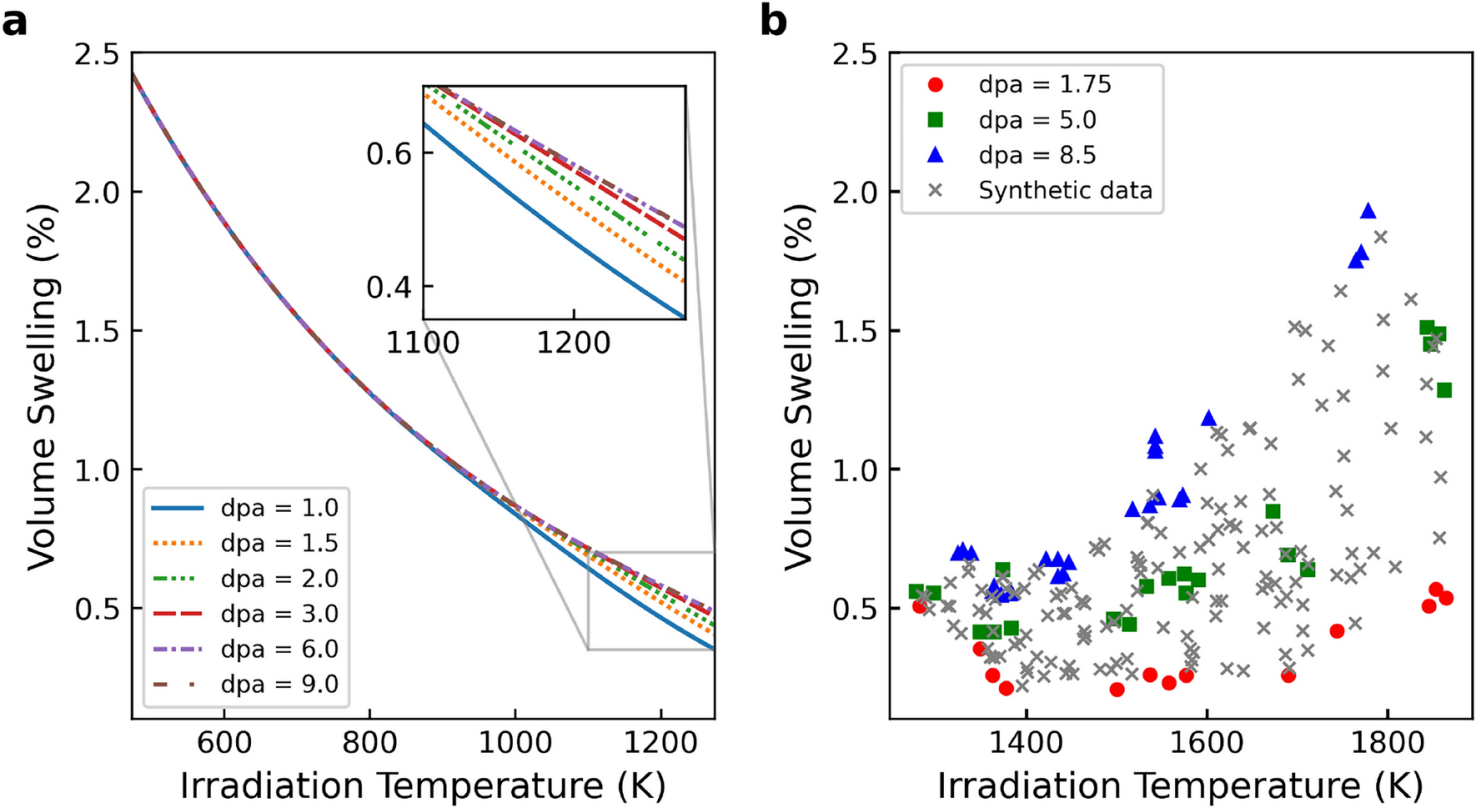
(**a**) Katoh model prediction of SiC volumetric swelling versus temperature and neutron fluence quantified in displacement per atom (dpa). Up to approximately 800 K irradiation temperature, dpa has no effect on volume swelling, but above that temperature, the effect becomes more pronounced (i.e., point defect). (**b**) CVD SiC volumetric swelling experimental data combined by Ref.^14^. Only a part of the synthetic data generated in this work is depicted for visibility here.

The Katoh model was empirically formulated to describe swelling in CVD SiC and SiC/SiC as a function of irradiation temperature and neutron fluence^12,13^. It was developed by^13^ using an extensive experimental database on swelling in CVD SiC and SiC/SiC. The dataset included historic swelling results from multiple studies on high-purity CVD SiC irradiated at temperatures ranging from 473 to 1073 K. This collected dataset allowed correlation of swelling with both irradiation temperature and fluence over a wide range encompassing the point defect and void swelling regions. Reference^13^ derived the mathematical form of the model (Eq. 1) based on theoretical considerations of point defect accumulation and swelling saturation mechanisms.

1 $$\begin{aligned} S = S_s\left[ 1-\exp \left( \frac{\gamma }{\gamma _c} \right) \right] ^{2/3}, \end{aligned}$$

where the critical dose $\gamma _c$ and saturable swelling $S_s$ are:

2 $$\begin{aligned} \gamma _c= & -0.57533+3.3342\times 10^{-3}T-5.3970\times 10^{-6}T^{2}+2.9754\times 10^{-9}T^{3}, \end{aligned}$$

3 $$\begin{aligned} S_s= & 5.8366\times 10^{-2} - 1.0089 \times 10^{-4}T + 6.9368\times 10^{-8}T^{2} - 1.8152\times 10^{-11}T^{3}, \end{aligned}$$

where *S*, $\gamma$, and *T* denote swelling (%), displacement damage per atom (dpa), and temperature (K), respectively. The model is validated on irradiation temperatures from 473 to 713 K. Figure 1a shows the model calculation with several dpa values. As the author^13^ pointed out, the model shows agreement with previously published swelling data for CVD SiC and CVI SiC/SiC at temperatures up to 1073 K. In higher-temperature regions, the decrease in swell is slower, but the influence of dpa on the change is evident. As the dpa increases, the decrease in the swelling is mitigated. However, as Fig. 1b shows, the model can not describe void swelling phenomena as shown in experimental data reported in Ref.^14^. Although the applicable temperature of this model is sufficient for conventional light water reactor operation, the current model is insufficient for future power increases of nuclear reactors, core design such as fast reactors, or estimation of accident scenarios such as loss of coolant accident (LOCA). Against this background, there is a need in the field of nuclear engineering to expand the limitations of existing models.

In order to extend an existing empirical model, gathering experimental data under new conditions such as irradiation temperature and neutron fluence is necessary. However, obtaining new data poses experimental challenges. Irradiation experiments require specialized facilities like research reactors or particle accelerators with limited availability and high utilization costs. Test setup and instrumentation to provide desired environmental conditions and measure property changes further add complexity. Executing experiments to accumulate sufficient data across a wide range of temperatures, dpa, and compositions relevant to nuclear applications is tremendously time-consuming. These experiments often take months to years, from design to analysis. Moreover, effects like swelling accumulate over a long duration, necessitating lengthy irradiation. The expense and effort to generate comprehensive experimental data represent a key bottleneck in developing predictive irradiation effects models.

### Physics-regularized neural network

**Figure 2:**
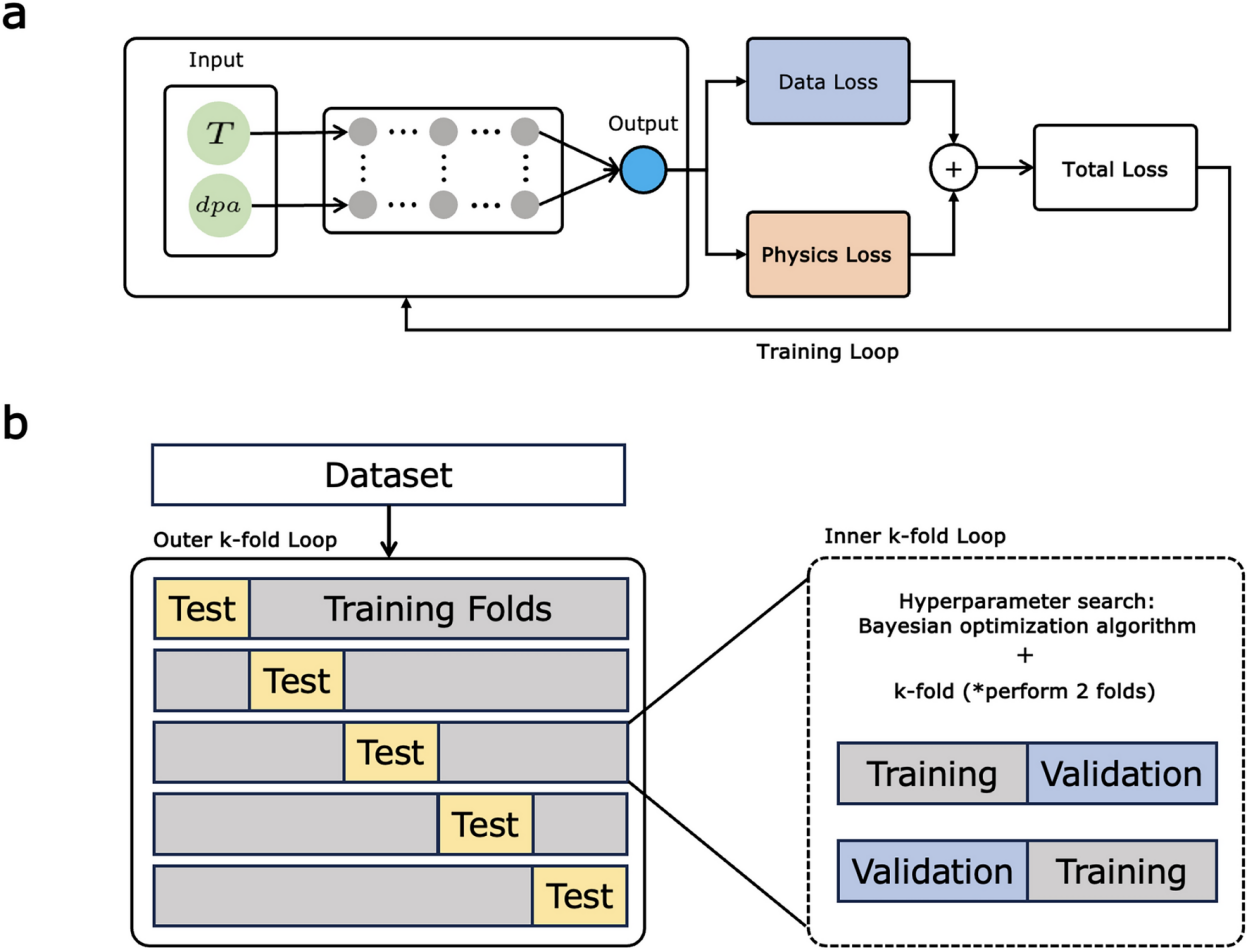
(**a**) Schematic of the physics-regularized neural network (PRNN) architecture comprising fully connected layers. (**b**) Overview of the nested cross-validation (NCV) procedure with inner loops for hyperparameter tuning and outer loops for ensemble model training and evaluation.

The extended volume swelling model was built with the proposed PRNN. The schematic of the proposed PRNN architecture is shown in Fig. 2a. This study employs a fully connected neural network (FCNN) with a dropout with one input layer, hidden layers, and one output layer. Conventional NNs only use an output from an output layer to compute a loss function during training. However, the proposed method is utilized to compute the loss value and to obtain an additional quantification. The loss value of the proposed PRNN comprises three terms: data loss, physics regularization, and L2 regularization.

For an input vector $x \in \mathbb {R}^{n}$, let $y_{i}$ be the true target value and ${\hat{y}}_{i}$ be the model output for the *i*-th training data point, where *i* indexes over the *N* total training points. The data loss term measures the mean-squared error (MSE) between the predicted ${\hat{y}}_{i}$ and true $y_{i}$ values over all training data points:

4 $$\begin{aligned} \text {Data loss} = \frac{1}{N}\sum _{i=1}^{N}(y_{i} - {\hat{y}}_{i})^2. \end{aligned}$$

By minimizing this MSE loss, the model is trained to make predictions ${\hat{y}}_{i}$ that are as close as possible to the true targets $y_{i}$, averaged over the *N* training data points.

The proposed model uses a physics regularization term in the loss function to integrate scientific domain knowledge into the training process for improved generalization and extrapolation. This term serves multiple objectives. It encodes consistency with established physical principles and new experiment data. Penalizing deviations from known behavior prevents unphysical predictions while guiding the model in line with validated relationships. It also supplements data-driven learning with scientific priors to compensate for limited training data. Moreover, the physics-based loss allows encoding specific constraints as needed, such as enforcing agreement with empirical swelling models under certain conditions. This term enables flexible integration of domain knowledge, only applying principles where reliably known. This characteristic maintains a blend of data-driven flexibility and scientific consistency even when new data is sparse.

Let $N$ represent the total number of training data points. The physics regularization term is applied to each data point $i$ based on a temperature threshold. Specifically, within the training set ${\mathcal {D}}$, the physics regularization term is defined as:

5 $$\begin{aligned} \text {Physics Regularization} = \frac{\lambda _{\text {phys}}}{N}\sum _{i=1}^{N} R_{i}^{2}, \end{aligned}$$

where $\lambda _{\text {phys}}$ is the physics regularization hyperparameter, and $R_{i}$ represents the residual between the model output ${\hat{y}}_{i}$ and the physics model output $y_{\text {phys},i}$, as given by:

6 $$\begin{aligned} R_{i} = {\left\{ \begin{array}{ll} {\hat{y}}_{i} - y_{\text {phys},i} & \text {if } T_{i} < 713 \, \text {K},\\ 0 & \text {otherwise.} \end{array}\right. } \end{aligned}$$

In addition to the data loss and physics regularization terms, the model loss function includes an L2 regularization term:

7 $$\begin{aligned} \text {L2 Regularization} = \lambda _{reg}\sum _{k=1}^{L}w_k^2, \end{aligned}$$

where $w_k$ are the weight parameters of the neural network model, $\lambda _{reg}$ is the L2 regularization hyperparameter, and the summation is over all *L* weight parameters $w_k$. L2 regularization reduces model complexity and sensitivity to training data variations by preventing the weights from reaching excessive values. The roles of these terms in the proposed method are to avoid overfitting, enhance numerical stability during training, and allow the integration of physics terms without dominating training.

By combining Eqs. (4), (5), and (7), the total loss to be used during training is expressed as follows:

8 $$\begin{aligned} Loss = \underbrace{\frac{1}{N}\sum _{i=1}^{N}(y_{i} - {\hat{y}}_{i})^{2}}_{\text {Data Loss}} + \underbrace{ \frac{\lambda _{phys}}{N}\sum _{i=1}^{N}R_{i}^{2}}_{\text {Physics Regularization}} + \underbrace{\lambda _{reg}\sum _{k}^{L}w_{k}^{2}}_{\text {L2 Regularization}}. \end{aligned}$$

### Training and cross-validation

The PRNN model training aims to minimize the composite loss function defined in Eq. (8). Optimization is performed using stochastic gradient descent via the Adam algorithm^29^. At each training iteration, it computes the gradient of the loss with respect to model parameters based on a randomly sampled batch of training data. Model weights are then updated in the direction that reduces the loss, iteratively converging to a minimized loss value. During the training process, the number of epochs is fixed to 100. Other hyperparameters, including learning rate and batch size, are optimized for cross-validation to ensure stable and efficient training dynamics given the data constraints.

In this study, the development of ML models is confronted by the limitations of a small dataset, which causes the inability to establish a comprehensive training and testing framework and increases the risk of model overfitting. The sparsity of data also demands a methodological approach that can provide an unbiased assessment of the ML models’ performance. NCV^30,31^ is employed to meet these challenges, optimizing the use of limited data via a stratified two-level cross-validation process. Specifically, the NCV structure is composed of a fivefold outer loop for the final model performance assessment, coupled with a twofold inner loop dedicated to hyperparameter optimization, resulting in a total of 5 outer folds $\times$ two inner folds as illustrated in Fig. 2b.

**Figure 3:**
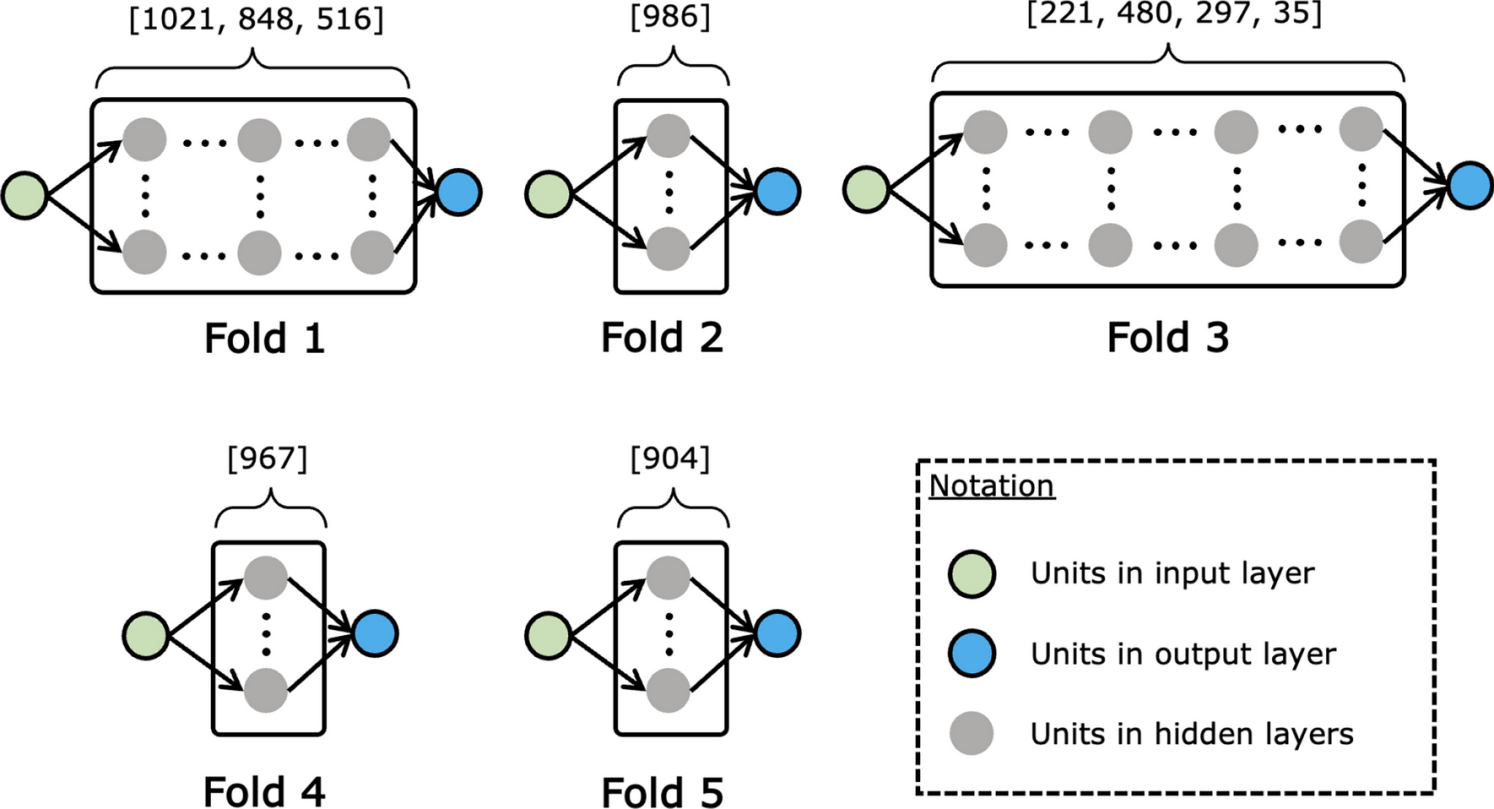
Model architectures of physics-regularized neural networks (PRNNs) for swelling prediction, determined through fivefold NCV. Numerical annotations indicate the number of units in fully connected layers, each employing ReLU activations. Each layer features a dropout mechanism with a specified rate, denoted as ‘Droprate ’.

Within the inner loop, the training data for each outer fold is split into two distinct subsets via a twofold cross-validation (CV). One subset functions as a validation set, while the complementary subset is utilized for model training across varying hyperparameters. This inner validation cycle is iteratively conducted for each of the five outer folds (see Fig. 3) to refine hyperparameters tailored to the dataset of the respective outer fold. Conversely, the outer loop employs a fivefold CV to partition the dataset into five separate subsets. Subsequently, for each iteration of the outer fold, an ensemble model is trained on the aggregate of the remaining four subsets, applying the hyperparameters refined in the inner loop. This process yields five independently tuned ensemble models corresponding to the five outer folds. The reserved outer test sets serve as instruments for an unbiased final evaluation.

The deployment of NCV enables the full exploitation of the constrained dataset for model calibration and validation, thereby assuring that the model’s performance reflects its predictive capabilities on unseen data. Hence, the application of NCV transcends methodological preference, becoming a compelled strategy due to the data limitations inherent in this study.

### Hyperparameter tuning in NCV

A key advantage of NCV is the ability to extensively search for an optimal combination of hyperparameters that maximize model performance. Table 1 summarizes the hyperparameters and search space explored through the inner folds of the NCV routine.

The physics weighting parameter $\lambda _{phys}$ determines the balance between data-driven and physics-based loss terms. The L2 regularization strength $\lambda _{reg}$ controls model complexity. Neural network architecture is tuned by varying the number of layers from 1 to 4 and the number of units in each layer. Additional hyperparameters like dropout rate, learning rate, batch size, and activation functions—with a choice between hyperbolic tangent and rectified linear unit (ReLU) functions—are also optimized. For efficient Bayesian optimization over the defined search space, the tree-structured Parzen estimator (TPE) algorithm is employed^32–35^. The objective function for tuning is the NCV inner loop validation loss. One hundred hyperparameter configurations are sampled to find the setting minimizing validation error for each inner loop.

Table 2 provides the best hyperparameters selected for each of the five outer fold models through this NCV-based search. The ReLU activation function is the preferred choice across all outer loops. The optimized configurations vary across folds, demonstrating the importance of fold-specific tuning. Full hyperparameter optimization details are available in the Methods section.

**Table 1:** List of hyperparameter search space used in NCV.

Hyper parameter	Type	Search space
$\lambda _{phys}$	Float	[0.001, 1.0)
$\lambda _{reg}$	LogUniform	[0.00005, 0.1)
Droprate	Float	[0.0, 0.5)
Learning rate	LogUniform	[0.00005, 0.1)
Batch size	Categorical	[16, 32, 64, 128]
Number of layers	Int	[1, 4]
Units in 1st layer	Int	[32, 1024]
(Units in 2nd layer)	Int	[32, 1024]
(Units in 3rd layer)	Int	[32, 1024]
(Units in 4th layer)	Int	[32, 1024]
Activation function	Categorical	‘Tanh’ or ‘ReLU’

**Table 2:** Hyperparameters determined through nested cross-validation.

Fold	Layers/units	$\lambda _{\text {phys}}$	$\lambda _{\text {reg}}$	Droprate	Learning rate	Batch size
1	[1021, 848, 516]	$3.61 \times 10^{-1}$	$2.72 \times 10^{-5}$	$3.80 \times 10^{-4}$	$1.02 \times 10^{-5}$	16
2	[986]	$8.20 \times 10^{-1}$	$1.39 \times 10^{-4}$	$1.17 \times 10^{-2}$	$1.82 \times 10^{-4}$	16
3	[221, 480, 297, 35]	$3.66 \times 10^{-1}$	$1.60 \times 10^{-5}$	$3.18 \times 10^{-4}$	$3.51 \times 10^{-5}$	16
4	[967]	$4.89 \times 10^{-2}$	$4.16 \times 10^{-5}$	$4.34 \times 10^{-1}$	$4.15 \times 10^{-4}$	32
5	[904]	$3.09 \times 10^{-1}$	$9.27 \times 10^{-5}$	$9.46 \times 10^{-2}$	$3.18 \times 10^{-4}$	32

### Swelling model via PRNN

**Figure 4:**
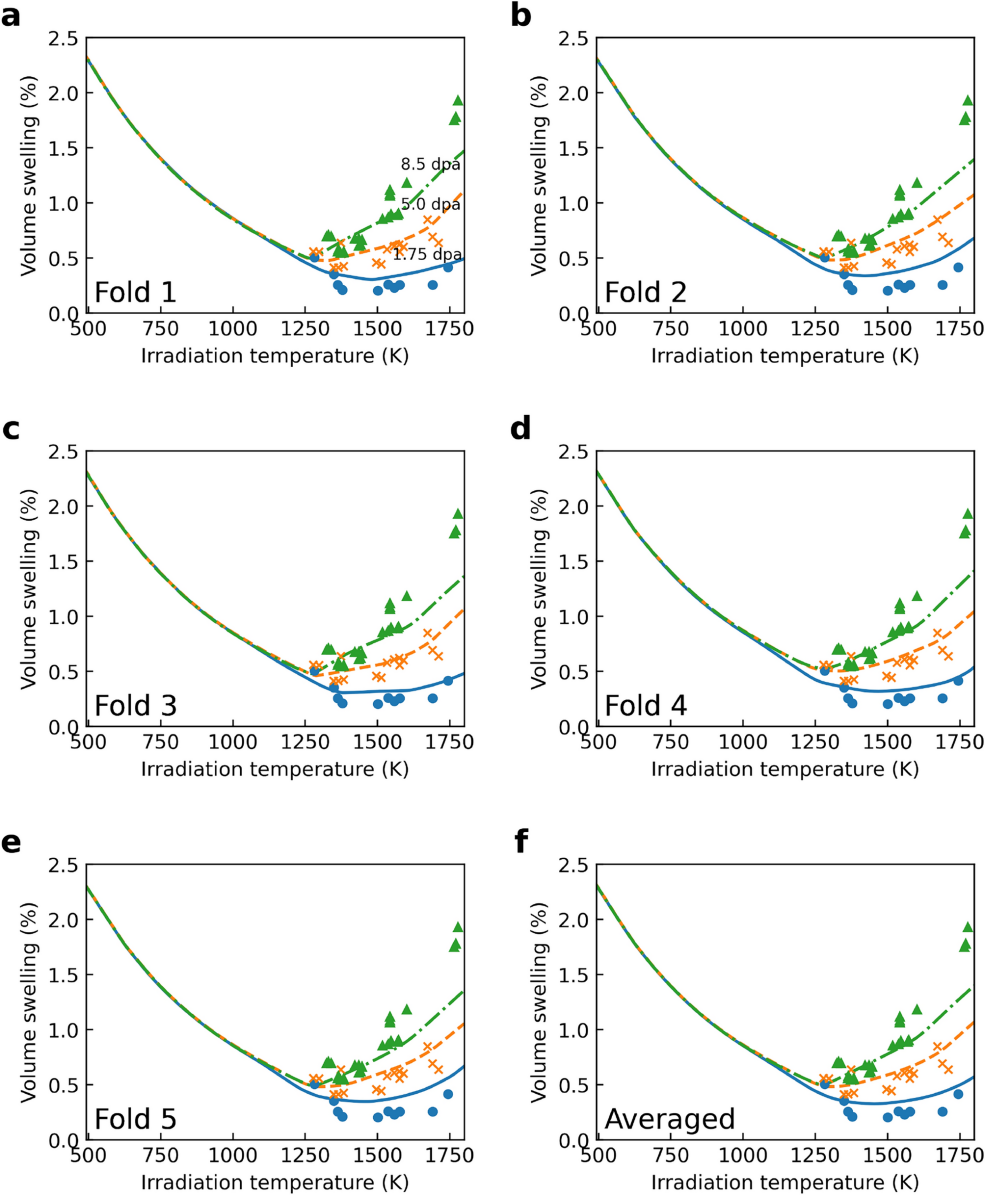
(**a–e**) Individual volume swelling predictive models generated across each cross-validation fold via the physics-regularized neural network (PRNN). Markers represent experimental data, lines show predictions. Models display consistency in capturing distinct point defect and void swelling regimes. (**f**) Aggregated swelling model obtained by ensembling the predictions of the models in (**a–e**). The final model accurately describes the full temperature range, aligning well with measured data for all irradiation doses. The ensembling demonstrates improved generalization ability versus individual models.

The PRNN approach to predict the volumetric swelling of SiC due to irradiation is divided into five distinct volume swelling models, as seen in Fig. 4a–e. Each model’s unique architectural and hyperparameter makeup was refined through NCV, ensuring accuracy and robustness.

Figure 4 demonstrates the models’ capability to capture the varying swelling behaviors across different temperature regimes. Notably, at temperatures below approximately 1000 K, swelling exhibits little to no dependence on irradiation dose, indicative of the point defect swelling regime. In contrast, above 1200 K, the models show a dose-dependent swelling trend, characteristic of the void swelling regime, where higher doses lead to greater volume expansion.

A swelling model was derived by averaging the predictions from these individual PRNN models and is illustrated in Fig. 4f. This model characterizes the swelling behavior over the entire temperature spectrum, encompassing all irradiation doses. It confirms the PRNN’s adaptability to diverse irradiation conditions.

**Figure 5:**
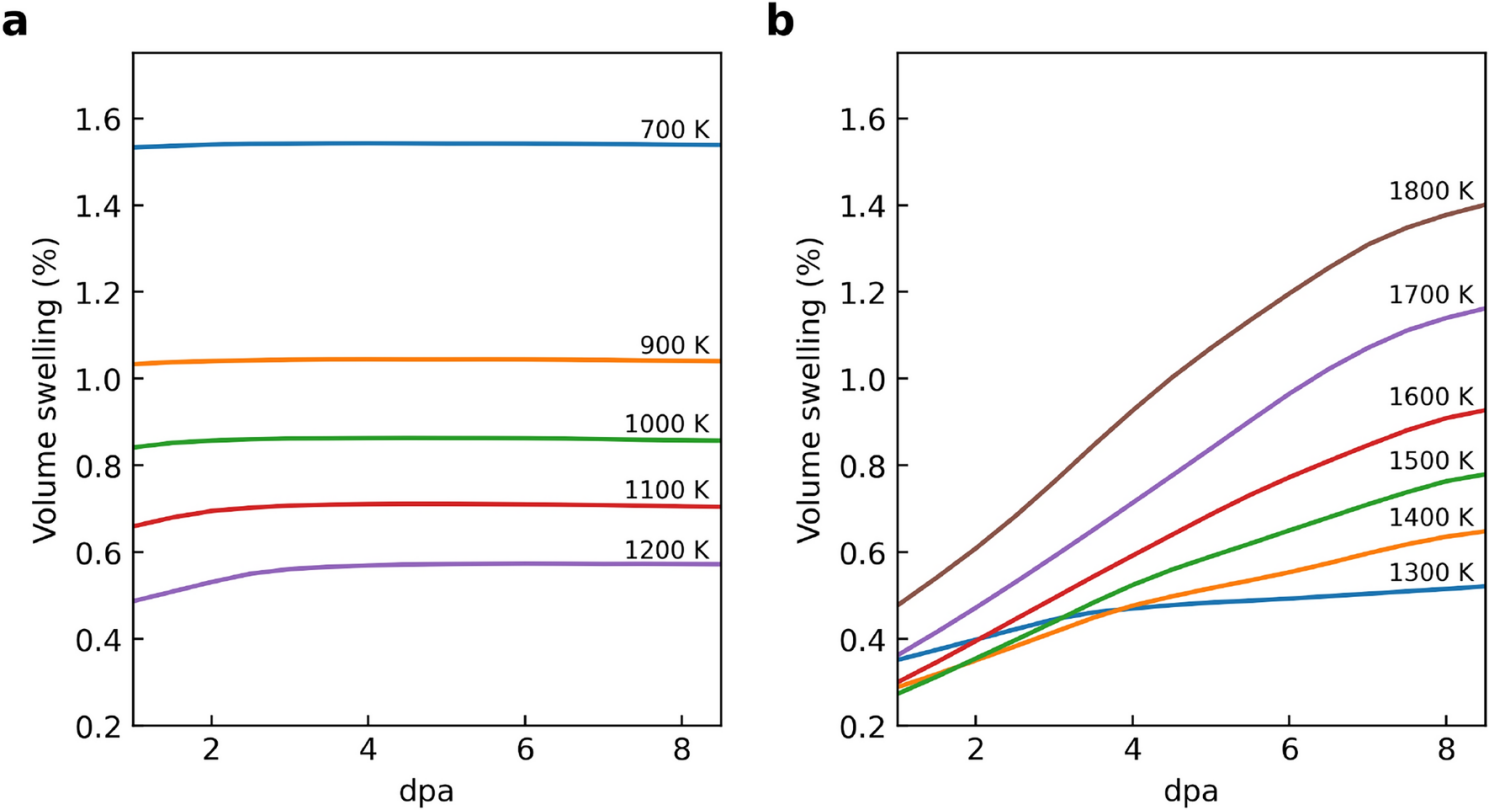
Dose-dependent swelling predictions by PRNN models across different temperature regimes. Panel (**a**) illustrates the relationship between displacements per atom (dpa) and volume swelling for irradiation temperatures less than 1200 K, highlighting the model’s predictions under lower temperature conditions. Panel (**b**) depicts a similar relationship for temperatures greater than 1300 K, showcasing the model’s performance under higher temperature conditions. The graphs in both panels demonstrate the PRNN model’s capability to accurately capture the swelling behavior of SiC across a range of irradiation doses and temperatures, effectively distinguishing between different swelling regimes.

Figure 5 enhances our understanding by presenting the relationship between dpa and volume swelling across two distinct temperature regimes. Panel (a) of Fig. 5 focuses on swelling at irradiation temperatures below 1200 K, while panel (b) examines temperatures above 1300 K. These visualizations provide an assessment of the PRNN model’s performance across various temperature ranges, offering insights into the responce of SiC to irradiation. The distinction between lower and higher temperature regimes is crucial, as it highlights the model’s accuracy and reliability in predicting swelling behavior under varying operational conditions.

Quantitative assessments, detailed in Table 3, further validate the PRNN models’ predictive accuracy. Test mean squared error values, ranging from $1.17 \times 10^{-2}$ to $1.43 \times 10^{-2}$, confirm to the model’s robust generalization capabilities, far beyond mere fitting to the training data.

In summary, the PRNN models’ extension of the empirical swelling relationship to higher temperatures, utilizing sparse experimental data, marks a advancement. Integrating physics-based constraints and data-driven learning within these models is important for expanding predictive capabilities.

**Table 3:** Best average validation and test scores from nested cross-validation for each fold, where hyper parameter tuning in an inner loop and the total loss function defined by Eq. (8).

Fold	Validation	Test	
	Custom loss	Custom loss	MSE
1	$1.27 \times 10^{-2}$	$1.38 \times 10^{-2}$	$1.38 \times 10^{-2}$
2	$1.33 \times 10^{-2}$	$1.20 \times 10^{-2}$	$1.20 \times 10^{-2}$
3	$1.31 \times 10^{-2}$	$1.32 \times 10^{-2}$	$1.32 \times 10^{-2}$
4	$1.36 \times 10^{-2}$	$1.17 \times 10^{-2}$	$1.17 \times 10^{-2}$
5	$1.28 \times 10^{-2}$	$1.43 \times 10^{-2}$	$1.42 \times 10^{-2}$

## Conclusions

This study presents an approach in the form of a PRNN for predicting the swelling in SiC due to irradiation. The PRNN model demonstrates an advancement over traditional empirical models, especially in the high-temperature domain, which is critical for applications in harsh irradiation environments like nuclear reactors and aerospace applications.

A key feature of the PRNN model is its ability to integrate empirical data with sparse experimental measurements, thus overcoming the limitations of existing models that struggle with high irradiation temperatures. The model’s architecture, empowered by physics-based regularization, allows for more robust predictions and helps in guiding extrapolation efforts. It is particularly important in SiC, where the accurate prediction of swelling behavior is crucial for ensuring material integrity and performance under extreme conditions.

The use of NCV in the model development process addresses the challenge of limited data availability, a common issue in high-stakes environments like nuclear engineering. The NCV minimizes the risk of the model overfitting to the limited data and simultaneously ensures the models’ accuracy in unseen scenarios. Furthermore, implementing hyperparameter optimization through Optuna adds another layer of precision, enabling the fine-tuning of the model to achieve optimal performance.

This study’s findings have an implications for advancing materials science, particularly nuclear engineering. The PRNN model’s ability to predict SiC swelling under various irradiation conditions with high accuracy enhances our understanding of material behavior under extreme conditions and opens up new avenues for developing more robust materials capable of withstanding harsh environments.

In conclusion, the integrated approach combining empirical knowledge, advanced machine learning techniques, and careful model tuning provides a pathway for enhancing predictive modeling in materials science. While the study focuses on SiC, the methodology, and insights gained can be applied to other materials and scenarios, broadening the scope of this research.

## Data Availability

The data that support the findings of this study are available within the article. Additional simulation outputs and neural network predictions supporting the findings are available from the corresponding author upon reasonable request.
